# The Performance of SARS-CoV-2 Serology Testing in the Diagnosis of COVID-19

**DOI:** 10.30699/ijp.2021.526032.25971

**Published:** 2021-08-05

**Authors:** Alireza Abdollahi, Samaneh Salarvand, Vahid Mehrtash, Bita Jafarzadeh, Mohammadreza Salehi, Reza Ghalehtaki, Saeed Nateghi

**Affiliations:** 1 *Department of Pathology, Imam Khomeini Hospital Complex, Tehran University of Medical Sciences, Tehran, Iran*; 2 *Department of Infectious Diseases and Tropical Medicine, Tehran University of Medical Sciences, Tehran, Iran*; 3 Radiation Oncology Research Center, Cancer Institute, Tehran University of Medical Sciences, Tehran, Iran; 4 Department of Cardiology, Tehran University of Medical Sciences, Tehran, Iran

**Keywords:** Antibody, COVID-19, SARS-CoV-2, Serology

## Abstract

**Background & Objective:**

COVID-19 reverse transcription-polymerase chain reaction (RT-PCR) has been a reference test for diagnosing a disease since the very beginning of the pandemic. COVID-19 serology tests have also been developed and used to estimate the prevalence of individuals who have already been infected. We aimed to evaluate the performance of serology tests for the diagnosis of patients who had been referred to medical centers with acute symptoms.

**Methods:**

In this cross-sectional study, 80 individuals suspected of COVID-19 who had been referred to Imam Khomeini Hospital Complex, Tehran, Iran, were examined. Upper respiratory tract specimens for RT-PCR and blood samples for COVID-19 IgM and IgG antibody level tests were collected and the results were compared.

**Results:**

The overall proportion in agreement, the agreement between positive results, and the agreement between negative results when comparing RT-PCR and IgM serology test were 40% (kappa = -0.006, *P *= 0.9), 32%, and 66.6%, respectively, and when comparing RT-PCR and IgG serology test were 46% (kappa = -0.006, *P *= 0.94), 43.5%, and 55.5%, respectively.

**Conclusion:**

The absence of a gold standard method for the diagnosis of COVID-19 makes it very challenging to determine the true sensitivity and specificity of different methods. The study results revealed no agreement between the two methods; so the RT-PCR test for upper respiratory tract specimen cannot be replaced with COVID-19 serology test for the diagnosis of patients with acute symptoms.

## Introduction

We have faced a novel viral respiratory infection since about one year ago (1). Although it seems we have had enough time for developing an accurate and reliable diagnostic test for coronavirus disease 2019 (COVID-19), there were too many obstacles on the road (2). Early in the course of the outbreak, the genome mapping of severe respiratory syndrome coronavirus 2 (SARS-CoV-2) made the utilization of reverse transcription-polymerase chain reaction (RT-PCR) operational for detecting the viral RNA (3). The identification of infected individuals and the consecutive administration of appropriate clinical and preventive interventions are critical steps in controlling the spread of COVID-19 (4). The final decision regarding the COVID-19 status in suspicious individuals is made in the context of clinical and imaging findings alongside the results of the viral RNA test (5). Collecting naso- and oro-pharyngeal swabs from patients' upper respiratory secretions is the most common sampling method (6). False negative RT-PCR test results have been reported in 2 to 29% of cases based on repeated testing studies, which could be an inaccurate estimation of test sensitivity due to the limitations of such studies (7). Unfortunately, the absence of a gold standard diagnostic test for COVID-19 makes the clinical judgment and evaluation of a new test difficult. Serology tests for detecting antibodies against SARS-CoV-2 have been developed, but as explained above, it is practically impossible to answer the questions about their sensitivity and specificity definitely (8). The best we can do is to consider the available RT-PCR test as an arbitrary reference test, and calculate its positive and negative agreement against a new test (9). In the present study, we aimed to compare the results from the COVID-19 serology test with those from the RT-PCR test performed on naso- and oro-pharyngeal swabs, and evaluate the possibility of utilizing serology tests to detect COVID-19 in symptomatic patients.

## Material and Methods

In this cross-sectional study, adult individuals presented to Imam Khomeini Hospital Complex, Tehran, Iran, with symptoms indicative of coronavirus disease 2019 during the course of the study were examined. All patients demonstrated clinical impression of COVID-19 based on a series of symptoms, including fever, dry cough, sore throat, headache, shortness of breath, tiredness, gastrointestinal disturbances, and/or decreased sense of smell or taste, as well as radiological abnormalities based on the national guideline for the diagnosis and treatment of COVID-19 published by Iran Ministry of Health and Medical Education (10). Demographic data of the patients were extracted from their medical records and the interval between the onset of their symptoms and serology testing were inquired. Patients with a previous history of COVID-19 or any upper respiratory infection since the emergence of COVID-19 were excluded from the study. 

Naso- and oro-pharyngeal swab samples were collected from each patient, and both swabs were inserted into a single sterile transport tube containing the viral transport medium. The entire sampling procedure was performed by trained healthcare personnel supplied with the proper personal protective equipment under the relevant droplet and contact precautions regulated by the Iran Ministry of Health. Samples were used for RNA extraction utilizing the Viral Nucleic Acid Extraction kit provided by RBC Bioscience (Taipei, Taiwan), according to the manufacturer's protocols. RT-PCR was performed using the Novel Coronavirus (2019-nCOV) Nucleic Acid Diagnostic Kit (PCR-Fluorescence Probing) of Sansure Biotech (Changsha, China) on the CFX96™ Real-Time PCR Detection System (Bio-Rad Laboratories, Inc.) coupled with a thermal cycler according to the manufacturer's instructions. 

Simultaneously, blood samples that were taken for biochemical analysis were used to evaluate the status of SARS-CoV-2 antibodies. All tests were performed following the verbal informed consent obtained from the participants. The enzyme-linked immunosorbent assay-based SARS-CoV-2 IgG and SARS-CoV-2 IgM Capture tests were used that were provided by Pishtazteb (Tehran, Iran). Plasma collected from the patients was used for assay according to the manufacturer's instructions, and the cut off index (COI) of the samples was calculated, which was interpreted as negative (COI<0.9), positive (COI>1.1), and borderline (0.9 < COI <1.1) results. 

Data were entered to and analyzed using SPSS software version 26 (SPSS Inc., Chicago, IL, USA). Continuous variables were reported as mean ± standard deviation (SD) for normally distributed and median (IQR) for non-normally distributed data. Student t-test or Mann–Whitney U test were used to compare continuous variables where appropriate. For assessing the degree of concordance between the results obtained from RT-PCR tests and serology tests, proportion in agreements as well as Kappa statistics were calculated. To analyze differences between several independent groups, the Kruskal-Wallis test was used followed by Jonckheere-Terpstra trend test to look for a linear trend in data. P-values of less than 0.05 were considered statistically significant in the study. 

This study was performed after obtaining the ethical approval from the Ethics Committee of Tehran University of Medical Sciences, Tehran, Iran. 

## Results

In this cross-sectional study, 80 individuals were examined to investigate the role of antibody testing, including both IgG and IgM antibodies against SARS-CoV-2, in the diagnosis of COVID-19. The mean age of subjects was 57.8 ± 1.2 years. The sample included 43 (54%) men with a mean age of 54.9 ± 2.7 years, and 37 (46%) women with a mean age of 61 ± 2.6 years. Among the patients, 62 (77.5%) cases were tested positive and 18 (22.5%) cases were tested negative for COVID-19 by RT-PCR. The median (IQR) interval between the onset of symptoms and referral to the hospital was 8 days (5.75%).

In total, SARS-CoV-2 IgM and IgG antibody serology tests were positive in 26 (33%) and 35 (44%) patients, and negative in 54 (77%) and 45 (66%) patients, respectively. Twenty-one patients had positive results for both antibodies, 14 were only IgG positive, 5 were only IgM positive, and 40 patients were tested negative for both.

The Majority of positive results for SARS-CoV-2 serology tests were observed in patients who had manifested the symptoms more than seven days before the test (77% and 65% for SARS-CoV-2 IgM and IgG antibodies, respectively). The comparison of seroconversion time between IgM and IgG indicated that more individuals had earlier seropositivity for IgG than IgM, meaning that 35% of positive IgG cases were detected in their first seven days after symptoms versus 23% for IgM. 

Assuming SARS-CoV-2 RT-PCR test results as a reference for naso- and oro-pharyngeal swabs, diagnostic performance for COVID-19 serology tests and agreement between the two methods are depicted in Table 1. 

The median (IQR) IgG index in patients with positive and negative RT-PCR test results were 0.96 (3.98) and 1 (10.4), respectively (*P*= 0.6). While the median (IQR) IgM index in patients with positive and negative RT-PCR test results were 0.35 (1.58) and 0.1 (5.9), respectively (*P*= 0.9). The IgG and IgM index levels showed no statistically significant difference between the two groups with regards to RT-PCR test results.

**Fig. 1:**
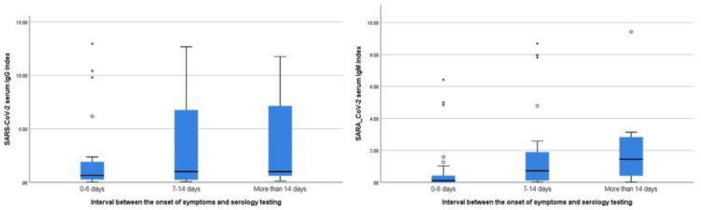
The COVID-19 serology antibody test results based on disease duration

Participants with positive RT-PCR test results were split into three independent groups that varied in the times at which the tests were performed after the onset of COVID-19 symptoms (Figure 1). SARS-CoV-2 IgG index values were not significantly influenced by the interval between the onset of symptoms and serology testing (*H* (2) =1.41,* P*=0.49). In contrast, SARS-CoV-2 IgM index values were affected significantly by the interval between the onset of symptoms and serology testing (*H* (2) =8.02, *P*=0.018). Mann-Whitney U tests were used to follow up this finding. A Bonferroni correction was applied and so all effects were reported at a 0.025 level of significance. The results indicated that IgM index values were significantly higher in COVID-19 cases tested 7 to 14 days after the onset of symptoms (U=229.5, r=0.33), and more than 14 days (U=43, r=0.36) after the onset of symptoms compared to the patients tested within the first 6 days of the onset of symptoms. The Jonckheere test showed a significant trend in the IgM and IgG index data, revealing that in COVID-19 cases the median IgM and IgG index values move in the same direction with the disease duration (IgM index value: *J*=781.5, Z=2.88, r=0.36; IgG index value: J=654, *Z*=1.16, r=0.15). 

**Table 1:** Agreement between the results of RT-PCR and serology tests in the detection of SARS-CoV-2 infection

SARS-CoV-2 RT-PCR	SARS-CoV-2 IgM serology									SARS-CoV-2 IgG serology								
	Days 0-7			More than 7 days			Overall			Days 0-7			More than 7 days			Overall		
	(+)	(-)	Total	(+)	(-)	Total	(+)	(-)	Total	(+)	(-)	Total	(+)	(-)	Total	(+)	(-)	Total
(+)	5	22	27	15	20	35	20	42	62	11	16	27	16	19	35	27	35	62
(-)	1	8	9	5	4	9	6	12	18	1	8	9	7	2	9	8	10	18
Total	6	30	36	20	24	44	26	54	80	12	24	36	23	21	44	35	45	80
Positive percent agreement (Sensitivity) (95% CI)	18.52% (6.30-38.08)			42.86% (26.32-60.65)			32.26% (20.94-45.34)			40.74% (22.39-61.20)			45.71% (28.83-63.35)			43.55% (30.99-56.74)		
Negative percent agreement (specificity) (95% CI)	88.89 (51.75-99.72)			44.44% (13.70-78.80)			66.67% (40.99-86.66)			88.89% (51.75-99.72)			22.22% (2.81-60.01)			55.56% (30.76- 78.47)		
Proportion in agreement	36%			43%			40%			53%			41%			46%		
Kappa (95% CI)	0.042			-0.077			-0.006			0.19			-0.21			-0.006		
P-value	0.6			0.5			0.9			0.1			0.08			0.94		

## Discussion

The results of the current study indicated that COVID-19 serology testing for both IgG and IgM antibodies are neither sensitive enough nor specific enough as a diagnostic tool for the diagnosis of patients with acute symptoms referred to medical centers. The results were obtained by taking SARS-COV-2 RT-PCR test as a reference method. 

In a study performed on suspected and confirmed COVID-19 patients, the overall concordance between the results of RT-PCR and SARS-CoV-2 antibody tests were reported to be 86.4%, a substantial degree of agreement. In our study, only suspected patients for COVID-19 were examined, and the RT-PCR and serology tests were performed simultaneously. In contrast to the results of the study conducted by Spicuzza *et al.*, our results showed no agreement between the results of PCR and antibody tests, with overall positive percent agreements of about 32% and 43% for IgM and IgG tests, respectively (11). The overall proportion in agreement between the two methods in our study was 40% (kappa = -0.006) and 46% (kappa = -0.006), indicating no concordance. The observed difference could be explained by the fact that seroconversion for SARS-CoV-2 antibody titers mostly occurs in patients who were tested more than 10 days after the onset of symptoms, while in the study of Spicuzza *et al.*, patients were tested after a median time of 18 days after the onset of symptoms (11, 12). In our study, serology tests were performed on patients with acute respiratory symptoms with a median of 8 days after the onset of symptoms, and the negative serology test results in cases with positive RT-PCR could turn positive, if follow-up serology tests were done.

A report regarding the sensitivity of the five commercially available SARS-CoV-2 antibody tests conducted on the samples taken from COVID-19 RT-PCR positive cases showed positive rates between 52.5% and 90.8% depending on the test manufacturer and the duration of symptoms before the sample was taken (13). In the current study, the highest positive agreement between the serology and RT-PCR test results was observed in cases tested for SARS-CoV-2 IgG antibody performed less than 7 days after the onset of symptoms, with a 53% of agreement. 

According to the findings of Zhao *et al.*, RT-PCR test was the most sensitive test in cases the samples from whom were collected within the first 7 days of onset of symptoms, showing a 66% sensitivity, while the antibody assays were positive in only 38.3% of the same cases. In their study, as the disease progressed, the sensitivity of RT-PCR test decreased and the sensitivity of serology tests increased (14-16). Our findings are in line with these findings, as we observed the least positive percent agreement between the results of RT-PCR and serology tests in the samples taken within the first 7 days of onset of symptoms. Only 18.5% (IgM) and 40.7% (IgG) agreement were observed between the test results, a fact that could be explained by the low sensitivity of serology test results accompanied by high sensitivity of RT-PCR tests in the same period, leading to the least agreement between the two methods. Therefore, during the earlier stages of COVID-19, more infected patients can be detected using RT-PCR tests compared to the serology tests. In these early stages, antibody response has not been initiated yet, which leads to a lower positive percent agreement between the two methods.

Cases tested 7 days after the onset of symptoms in our study showed 43% and 46% positive percent agreements between the RT-PCR and serology tests. In this period, the chance of detecting the virus using RT-PCR decreased, but the positivity of serology tests increased (17, 18). At this point, some infected patients may have negative RT-PCR test results, however, the chance of getting positive serology test results has been increased. As a result, despite the improvement in the performance of serology tests in this period, the concomitant decline of RT-PCR test sensitivity is probably the source of agreement between two methods which still remains low. 

Studies on pre-epidemic samples as negative controls have reported that COVID-19 serology test specificity is about 98% with a slight deviation which can be explained by the test method (19). In our study, the evaluation of specificity was not possible due to the lack of a diagnostic gold standard test, and also due to the use of samples from suspicious COVID-19 patients. However, considering RT-PCR test as a reference test, in IgM and IgG serology tests, the negative percent agreements were 88% in cases tested within 7 days of the onset of symptoms, and the percentages declined dramatically to 44% and 22%, respectively, in patients tested more than 7 days after the onset of symptoms. COVID-19 RT-PCR clinical sensitivity is at its best during the first five days following the onset of infection and decreases gradually over time (20). As mentioned earlier, unlike PCR, sensitivity of serology test increases over the course of the disease, so one of the possible explanations for such a low negative percent agreement between the two tests is that infected patients with negative RT-PCR test results could be tested positive if samples were collected in the first week of their illness accompanying with the increased proportion of positive serology tests among those tested in this period.

One of the limitations regarding this issue is the lack of a proper gold standard test to could come to a final interpretation about the sensitivity and specificity of different diagnostic methods challenging in COVID-19 cases. Performing multiple consecutive RT-PCR and serology tests on patients upon their admission and during the course of their disease could be an effective way to be more precise.

## Conclusion

We aimed to evaluate a COVID-19 serology test as a diagnostic tool in patients referred to a medical center with acute respiratory symptoms and other COVID-19-related symptoms, and see whether it could be replaced with RT-PCR. Our study showed there is no agreement between the results of these two methods and a COVID-19 RT-PCR cannot be replaced with a serology test as a diagnostic tool.

## Conflict of Interest

The authors have no conflicts of interest to declare for this study.
